# Examining the Intersection between Drivers of Disparities: Social Determinants and Stress Reactivity in African American Breast Cancer Survivors

**DOI:** 10.1158/2767-9764.CRC-25-0388

**Published:** 2026-03-30

**Authors:** Fatimata Sanogo, Junhan Cho, Melanie S. Jefferson, Trista A. Beard, Oluwole Adeyemi Babatunde, Kelsie Campbell, Mariana C. Stern, Bodour Salhia, Chanita Hughes Halbert

**Affiliations:** 1 Department of Population and Public Health Sciences, University of Southern California, Los Angeles, California.; 2 Hollings Cancer Center, Medical University of South Carolina, Charleston, South Carolina.; 3 Prism Health Care, Greer, South Carolina.; 4 Norris Comprehensive Cancer Center, University of Southern California, Los Angeles, California.; 5 Department of Cancer Biology, University of Southern California, Los Angeles, California.

## Abstract

Responses to social stressors are important drivers of cancer risk and outcomes; however, current studies focus on neighborhood-level social determinants of health (SDOH). We evaluated within-subject trajectories in stress reactivity (i.e., change in salivary cortisol) among 60 African American patients with breast cancer and examined the moderating effects of social stressors on cortisol responses. SDOHs were obtained by self-report and were included in linear mixed-effects regression models that were generated for cortisol levels, including time (primary regressor) adjusting for time-invariant covariates. Significant increases in cortisol levels were observed at 12- and 24-minute post-Trier Social Stress Test tasks. Significant interactions between time and SDOHs on cortisol were identified. Higher and escalating stress reactivity was observed in patients with greater financial strain, social isolation, negative life events, and perceived stress. Social risk factors moderated stress reactivity; however, additional research is needed to understand the effects of physiologic stress reactivity on tumor etiology and progression, especially among patients who have greater exposure to social stressors.

**Significance:**

Chronic stress exposure promotes breast cancer initiation and progression; however, current studies focus on neighborhood-level deprivation even though individual variability in stress reactivity is an important driver of cancer risk and outcomes, especially among those who experience the greatest burden of disease. Self-reported data on social risk factors can be used to enhance the precision of cancer care by identifying patients who should be prioritized for referral to socially informed care and who are likely to experience dysregulated cortisol response to SDOHs.

## Introduction

Social determinants of health (SDOH) are important drivers of disease risk and health care outcomes. Accordingly, health care systems are required to screen patients for social risk factors as part of health equity quality metrics (1, 2). This focus on SDOH screening is relatively new, and best practices for SDOH screening and referral processes are emerging. For instance, neighborhood deprivation derived from geographic information may be used as a first step to screen patients for SDOHs because residential risk factors (3) can be characterized using data stored in the electronic health record (EHR; ref. 4). Because area-level data do not have sufficient precision to identify patients who should be prioritized for SDOH referral, information on self-reported social risk factors should be used for SDOH screening and referral to public health agencies and social service resources. At the same time, previous research has shown that social service and public health organizations have limited capacity to accept referrals from health care systems (5–7). Similarly, health care systems may not complete SDOH screening and referral using actionable and sustainable strategies.

One approach to enhance the precision and impact of SDOH screening and referral is to focus these efforts on identifying risk factors and unmet needs that are also associated with biological mechanisms important to disease processes, play a role in the utilization of health care services, and affect cancer care outcomes. The hypothalamic-pituitary-adrenal (HPA) axis, for instance, is the primary stress response pathway through which cortisol is released in response to stress exposure; findings from animal studies have shown that dysregulation of the HPA axis response is one biological mechanism through which social stressors contribute to breast cancer risk and outcomes (8). Studies have also demonstrated that glucocorticoids play a pivotal role in creating the cellular and molecular environments that are associated with outcomes and prognoses [e.g., cancer metastasis (9, 10) and reduced treatment efficacy; ref. 10] that contribute to racial disparities in breast cancer risk and outcomes. For instance, chronic exposure to stress hormones (e.g., cortisol) through the HPA axis is an important precursor to immune mechanisms that play a role in breast cancer development, progression, and response to treatment (11). At the same time, social isolation has been associated with breast cancer progression using animal models (8), and the out-of-pocket expenses for cancer care may reduce the quality of survivorship, especially among patients who experience financial strain before and during their diagnosis, treatment, and recovery (12).

Together, these findings suggest that multilevel social stressors—spanning individual (e.g., perceived stress), interpersonal (e.g., social isolation), and structural (e.g., neighborhood deprivation) levels—activate or dysregulate HPA axis responses, leading to altered cortisol reactivity and downstream biological processes relevant to breast cancer outcomes. However, empirical data testing the association between SDOH risk factors and cortisol responses among the patient populations who have greater exposure to these risk factors are not available. Prior studies have shown that African American women are more likely than other populations to live in geographic areas that have high levels of social deprivation (13), and our previous research has demonstrated that African American women and those who live in geographic areas that have high levels of social deprivation have an increased likelihood of advanced-stage disease at diagnosis (14). Recent national data continue to show disparities in breast cancer morbidity and mortality among African American women (15). Stress reactivity, or individual responses to stressful situations, could provide important insights about the biological impact of exposure to chronic social stressors while also generating empirical data to inform and enhance SDOH screening and referral practices in health care settings. For this reason, we examined within-subject variation in stress reactivity among African American patients with breast cancer by assessing cortisol levels over time in a laboratory-based stress reactivity study (16). We also examined the moderating effects of specific social stressors (e.g., social isolation, financial strain, and perceived stress) to determine whether greater exposure to these stressors increased physiologic stress reactivity during acute stress exposure among these patients. We hypothesized that Black breast cancer survivors exposed to higher SDOH will demonstrate altered (blunted or heightened) cortisol reactivity following the Trier Social Stress Test (TSST).

## Materials and Methods

### Study design, inclusion, and exclusion criteria

The study design and procedures are reported in detail elsewhere (16, 17) and are summarized here (see Supplementary Fig. S1). Study participants were African American patients with breast cancer who were diagnosed with early-stage or locally advanced disease (up to stage IIIa), had completed primary surgical intervention, were between 21 and 75 years of age at the time of diagnosis, and were within 6 years of being diagnosed (i.e., diagnosed between 2013 and 2020). Participants were instructed to refrain from eating, drinking, smoking, or engaging in vigorous physical activity for at least 1 hour prior to the TSST. Women were excluded if they had a major psychiatric illness requiring psychotropic medication. Participants were also excluded if they were unable to provide saliva samples or participate in the TSST procedures. All TSST sessions were scheduled at approximately 3:00 pm to minimize diurnal variation in cortisol secretion. Because our focus was on acute stress reactivity rather than diurnal cortisol rhythms, participants served as their own control in the pre–post stress comparisons. Patients were recruited into the study from 2018 to 2022. Institutional Review Boards at the Medical University of South Carolina and the University of Southern California approved the study. Written informed consent was obtained from all participants, and the study was conducted in accordance with the ethical guidelines of the Belmont Report.

### Procedures

The recruitment procedures for this study were described in detail previously (17), and following enrollment, patients were invited to complete two laboratory visits to measure stress biomarkers (e.g., cortisol) and to examine reactivity to a laboratory-based stressor using the TSST. The TSST is a standardized method for inducing the HPA axis and stress responses in a laboratory setting (18). The TSST protocol includes tasks that reflect elements of social stressors (e.g., lack of control, possible threat, and social evaluation) that activate stress hormones through the HPA axis (19). In this study, the speech task was completed first and was followed by an arithmetic task after an initial acclimation period. The TSST followed the standard protocol with two rooms, one for acclimation and one for tasks, 5-minute speech and math portions, and three judges attended all sessions. All participants were scheduled for 3:00 pm lab visits. Collection of the baseline saliva sample (T1) for all participants was standardized at 3:16 pm, immediately following the 15-minute acclimation period, within a 15-minute grace period to mitigate for diurnal variations (18). Five saliva samples were collected as part of the TSST: the baseline sample (T1) was collected immediately after the 15-minute acclimation period. The second sample (T2) was obtained immediately before patients began the speech task, and the subsequent saliva samples were collected at 2 (T3), 12 (T4), and 24 minutes (T5) after the TSST tasks were completed. Saliva samples were collected by placing a Sarstedt synthetic swab under the tongue for 2 minutes to passively absorb drool. Following collection, the swab was returned to its designated collection tube, which was then sealed and placed in an ice-filled container to maintain sample integrity until the conclusion of the TSST visit. At the end of the visit, collected samples were frozen at −20°C (short term) and stored at −80°C (longer term) before assay. All samples were thawed and centrifuged before laboratory testing. Salivary cortisol from the TSST was assessed using a cortisol saliva luminescence immunoassay, with interassay coefficients of variation ranging from 3.3% to 5% across low-to-high-concentration quality control samples. TSST samples for each participant were assayed on the same date using standard procedures to minimize processing errors.

### Measures

Self-reported data on socioeconomic factors were obtained during the baseline telephone interviews using items from our previous research (20). Breast cancer stage and the month/year of diagnosis were abstracted from EHRs. Time since diagnosis was calculated based on the amount of time between completion of the baseline and the date of diagnosis. Social stressors were measured in terms of negative life events, perceived stress, social isolation, and financial strain. We used the Life Events Questionnaire (21) to quantify and evaluate participants’ life events over the prior year. Specifically, we asked patients to report events concerning their health, employment, finances, or crime/legal issues. Each event’s valence was then rated as positive or negative and its impact on their lives (ranging from no effect to a major effect). We calculated a negative event score by summing the impact ratings for events that were identified as negative (22). The four-item Perceived Stress Scale (PSS; ref. 23) measured perceived stress, and social isolation was assessed using the University of California, Los Angeles, three-item Loneliness Scale questionnaire (24). Cronbach α for the PSS and social isolation scales was 0.76 and 0.77, respectively. Financial strain was measured using one item that asked patients to indicate how much money they have left over at the end of the month (25).

### Data analysis

First, descriptive statistics were generated to characterize the study sample in terms of sociodemographics, clinical factors, and social stress exposures. Next, we estimated mean cortisol levels across the five time points in the TSST. To examine within-subject changes in cortisol levels before and after TSST tasks, we tested the association between time and cortisol levels in the primary analysis. We fit linear mixed-effects models (LMM) to account for the interdependence of repeated cortisol measurements nested within participants. The primary models included fixed effects for time (categorical; reference = T1) with a participant-level random intercept to account for individual variability in baseline cortisol levels. Random slopes for time were explored but not retained because of model convergence limitations given the sample size (*n* = 60) and five cortisol time points. Unadjusted (time regressor only) and adjusted [controlling for sociodemographic and other covariates (e.g., as fixed effects of between subjects)] LMMs were performed. We estimated unstandardized regression coefficients (*b*) with 95% confidence intervals (95% CI) for the continuous outcome of cortisol levels.

In addition, we tested the moderating effects of social stressors. Specifically, we modeled interaction terms of time × social stress in a series of models for each social stress variable. We categorized participants into high– and low–social stressor groups based on median splits for continuous social stress variables. Although using the median split to dichotomize continuous variables into groups is a standard practice, this approach may reduce statistical power and obscure identification of dose–response effects. For the interactions found to be significant, we performed additional *post hoc* tests to understand how cortisol levels changed over time within each group. We estimated within-subject changes in cortisol levels over time, stratified by the levels of each social stress moderator (e.g., high vs. low levels). The interaction term tests whether individuals with higher chronic stress exhibit different cortisol trajectories (increase and recovery patterns) compared with those with lower stress exposure. As complementary summary measures of stress reactivity, we calculated (i) the area under the curve with respect to increase (AUCi) to capture overall cortisol increase from baseline across the stress period and (ii) the peak cortisol level (maximum posttask cortisol). These secondary outcomes were compared between high– and low–stress exposure groups using linear regression models adjusted for the same covariates (see Supplementary Table S2). Missing data were handled within the mixed-model framework using restricted maximum likelihood, which accommodates missing repeated measures without listwise deletion. Because overall missingness was minimal (<1%), multiple imputation was also conducted as a robustness check, yielding consistent results. Statistical significance was set at 0.05 (two-tailed). Benjamini–Hochberg multiple test corrections were applied to two-sided *P* values to maintain 0.05 FDR (26). We conducted all analyses using SPSS version 30 (RRID: SCR_002865; ref. 27).

## Results

### Sample characteristics

A total of 110 patients were included in the study, and of these, 72 (65%) completed the prechallenge visit, and 60 completed the TSST. Table 1 shows the characteristics of patients who completed the TSST and were included in this analysis. Most patients were not married and had some college education. Forty-five percent of patients were employed, 53% reported an annual income of $35,000 or higher, and 50.8% reported financial strain. The mean age among participants was 50.9 (SD = 8.5) years. Clinically, 58% were diagnosed with breast cancer more than 2 years before study enrollment, and 53% were diagnosed with stage 1b or lower disease.

**Table 1.** Characteristics of study participants^a^.

Variable	*N* (%)/M (SD)
Age, year, M (SD)	59.4 (8.5)
Marital status, *N* (%)	​
Married	22 (37)
Not married	38 (63)
Education level, *N* (%)	​
≥Some college	46 (77)
≤High school	14 (23)
Employment status, *N* (%)	​
Employed	27 (45)
Retired	22 (37)
Not employed	11 (18)
Income level, *N* (%)^b^	​
≥$35,000	30 (53)
<$35,000	27 (47)
Time since diagnosis, *N* (%)	​
≤2 years	25 (42)
>2 years	35 (58)
Stage, *N* (%)	​
IIa or greater	28 (47)
Ib or lower	32 (53)
Financial strain, *N* (%)^c^	​
Yes	30 (50.8)
No	29 (49.2)
Social isolation, M (SD)^d^	4.0 (1.9)
Perceived stress, M (SD)^e^	6.0 (3.4)
Negative events, M (SD)^f^	4.0 (2.5)

a Participants *N* = 60.

b Available data from *N* = 57 respondents.

c Available data from *N* = 59 respondents.

d Range of responses for social isolation score (3–9); higher values reflect higher perceived isolation.

e Range of responses for perceived stress score (4–20); higher values reflect higher perceived stress.

f Range of responses for negative life events score (0–12); higher values reflect more negative life events reported within the last 12 months.

### Cortisol changes during stress exposure

**Figure 1.**
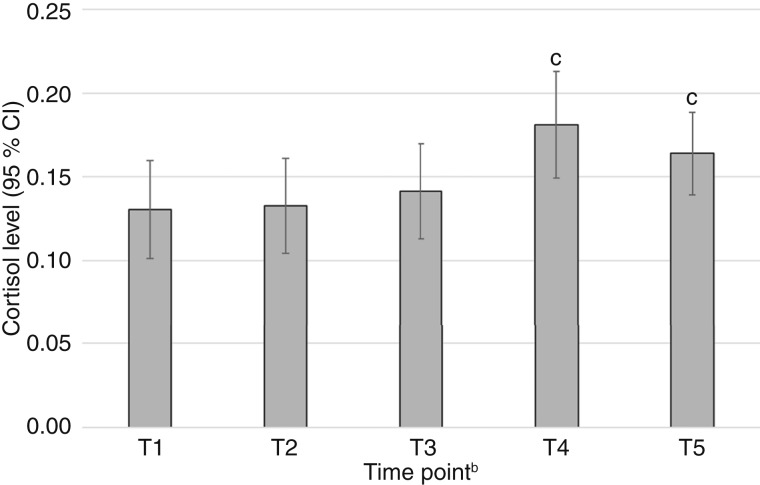
Mean cortisol levels over time^a^. ^a^, Analytic sample participants *N* = 60 (total observation *N* = 300). ^b^, T1 and T2 were assessed before TSST tasks and T3, T4, and T5 were assessed after TSST tasks. ^c^, Significantly different compared with T1 (baseline) in *post hoc* contrasts (*P* < 0.05).

Figure 1 shows the stress reactivity patterns across the repeated assessments during the TSST (T1–T5). The mean cortisol level at baseline (T1) among the overall sample was 0.13 (SD = 0.11), and there was no significant difference in mean cortisol levels between T1 and T2 (M = 0.13; SD = 0.11), suggesting stable levels of cortisol before TSST administration. Following TSST administration, mean cortisol levels began to increase at T3 (M = 0.14; SD = 0.11), reflecting the onset of an acute stress response, and reached a significant peak at T4 (12 minutes after TSST; M = 0.18; SD = 0.12; *P* = 0.01). Cortisol remained significantly elevated at T5 (24 minutes after task; M = 0.16; SD = 0.10; *P* = 0.04) relative to baseline. The observed peak increase of approximately 0.05 μg/dL corresponds to a moderate within-person effect (standardized β ≈ 0.35–0.40).

Table 2 shows within-subject changes in cortisol levels across time points [T1 (reference)–T5]. Compared with baseline (T1) levels, there were no significant increases in cortisol levels at T2 (b = 0.003; 95% CI = −0.003 to 0.010; *P* = 0.33) and T3 (b = 0.010; 95% CI = −0.009 to 0.030; *P* = 0.30) after adjusting for sociodemographic and social stress covariates. Compared with the T1 baseline cortisol level, there were significant increases in mean cortisol levels at T4 (b = 0.054; 95% CI, 0.026–0.082; *P* < 0.001) and T5 (b = 0.040; 95% CI, 0.015–0.065; *P* = 0.002). Associations of covariates with cortisol levels are presented in Supplementary Table S1.

**Table 2.** Associations between assessment time points and cortisol levels^a^.

Time^b^	Unadjusted model^c^		Adjusted model^d^	
	b (95% CI)	*P*	b (95% CI)	*P*
T1	Ref	—	Ref	—
T2	0.001 (−0.006 to 0.009)	0.72	0.003 (−0.003 to 0.010)	0.33
T3	0.011 (−0.008 to 0.029)	0.25	0.010 (−0.009 to 0.030)	0.30
T4	0.050 (0.023–0.077)	<0.001^e^	0.054 (0.026–0.082)	<0.001^e^
T5	0.038 (0.014–0.062)	0.002^e^	0.040 (0.015–0.065)	0.002^e^

Abbreviation: Ref, reference.

a Participants *N* = 60 (total observation points *N* = 300).

b T1 and T2 were assessed before the TSST tasks and T3, T4, and T5 were assessed after TSST tasks.

c Linear mixed-effect repeated-measures regression models for cortisol level outcome, including time with random intercept.

d Linear mixed-effect repeated-measures regression models for cortisol level outcome, including time with random intercept and time-invariant covariates (i.e., age, marital status, education level, employment status, income level, time since diagnosis, stage, financial strain, social isolation, negative life events, and perceived stress).

e Statistically significant after Benjamini–Hochberg corrections for multiple testing to control FDR at 0.05 (based on two-tailed corrected *P* value).

**Figure 2.**
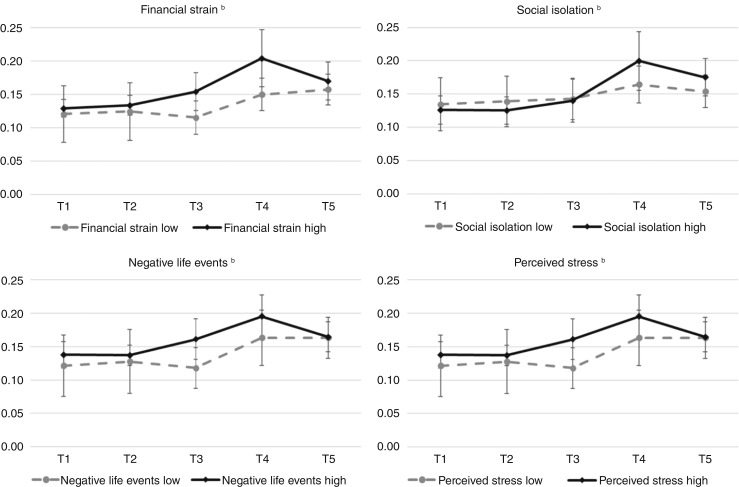
Mean cortisol levels across time points, stratified by social stress moderator statuses^a^. ^a^, Cortisol levels across time, stratified by moderator status (i.e., high vs. low). *Y*-axis = mean (95% CI) cortisol level. *X*-axis = time. ^b^, Each moderator (i.e., financial strain, social isolation, negative life events, and perceived stress) was coded as 0 (low) or 1 (high) using median split.

The results from the moderation analysis are reported in Table 3. Significant interactions were found for financial strain (*χ*^2^/df = 15.68/4; *P* = 0.003), social isolation (*χ*^2^/df = 11.51/4; *P* = 0.02), negative life events (*χ*^2^/df = 12.75/4; *P* = 0.01), and perceived stress (*χ*^2^/df = 11.40/4; *P* = 0.02). For all significant interactions, *post hoc* multigroup analyses showed consistent patterns in which there were no significant changes in cortisol levels across follow-ups (i.e., T2–T4), compared with T1, among patients who had lower exposure to social stressors whereas there were significant increases in cortisol levels at T4 and T5 (vs. T1) among patients who had greater exposure to social stressors (*P* values ≤ 0.007; see Fig. 2; individual cortisol level trajectories are presented in Supplementary Fig. S2).

**Table 3.** Interaction effect test^a^.

Interaction effect test^b^		
Interaction term	*χ* ^2^/df	*P*
Time X financial strain	15.68/4	0.003
Time X social isolation	11.51/4	0.02
Time X negative life events	12.75/4	0.01
Time X perceived stress	11.40/4	0.02

*Post hoc* multigroup analysis 1—financial strain^c^				
Time^d^	Low financial strain		High financial strain	
	b (95% CI)	*P*	b (95% CI)	*P*
T1	Ref	—	Ref	—
T2	0.002 (−0.004 to 0.009)	0.48	0.004 (−0.006 to 0.015)	0.41
T3	−0.005 (−0.028 to 0.018)	0.65	0.025 (−0.004 to 0.053)	0.09
T4	0.028 (−0.003 to 0.059)	0.08	0.075 (0.033–0.117)	<0.001^e^
T5	0.037 (−0.001 to 0.074)	0.06	0.041 (0.011–0.071)	0.007^e^

*Post hoc* multigroup analysis 2—social isolation^c^				
Time^d^	Low social isolation		High social isolation	
	b (95% CI)	*P*	b (95% CI)	*P*
T1	Ref	—	Ref	—
T2	0.004 (−0.003 to 0.012)	0.26	0.001 (−0.009 to 0.012)	0.78
T3	0.008 (−0.022 to 0.038)	0.59	0.012 (−0.010 to 0.033)	0.29
T4	0.030 (−0.005 to 0.065)	0.09	0.078 (0.038–0.118)	<0.001^e^
T5	0.019 (−0.018 to 0.056)	0.31	0.061 (0.034–0.088)	<0.001^e^

*Post hoc* multigroup analysis 3—negative life events^c^				
Time^d^	Low negative life events		High negative life events	
	b (95% CI)	*P*	b (95% CI)	*P*
T1	Ref	—	Ref	—
T2	0.005 (−0.005 to 0.014)	0.34	0.002 (−0.006 to 0.011)	0.56
T3	−0.004 (−0.029 to 0.022)	0.78	0.021 (−0.005 to 0.048)	0.11
T4	0.040 (−0.002 to 0.083)	0.07	0.062 (0.028–0.096)	<0.001^e^
T5	0.042 (0.000–0.083)	0.05	0.037 (0.010–0.063)	0.007^e^

*Post hoc* multigroup analysis 4—perceived stress^c^				
Time^d^	Low perceived stress		High perceived stress	
	b (95% CI)	*P*	b (95% CI)	*P*
T1	Ref	—	Ref	—
T2	0.001 (−0.008 to 0.008)	0.95	0.007 (−0.003 to 0.016)	0.18
T3	−0.005 (−0.028 to 0.018)	0.67	0.025 (−0.005 to 0.056)	0.10
T4	0.024 (−0.008 to 0.056)	0.14	0.081 (0.038–0.124)	<0.001^e^
T5	0.025 (−0.016 to 0.067)	0.23	0.054 (0.025–0.083)	<0.001^e^

a Participants *N* = 60 (total observation *N* = 300).

b Linear mixed-effect repeated-measures regression models for cortisol level outcome include time and the interaction term of time X each moderator (i.e., financial strain, social isolation, negative life events, and perceived stress), adjusting for time-invariant covariates presented in the adjusted model in Table 2. Each interaction effect for individual moderator was separately tested. For significant interaction terms based on the Wald *χ*^2^/df test, *post hoc* multigroup analyses were conducted to examine the association between time and cortisol level outcome, stratified by the moderator status.

c Each moderator was coded as 0 (low) or 1 (high) using median split.

d T1 and T2 were assessed before TSST tasks and T3, T4, and T5 were assessed after TSST tasks.

e Statistically significant after Benjamini–Hochberg corrections for multiple testing to control FDR at 0.05 (based on two-tailed corrected *P* value).

### Sensitivity analyses

To complement the primary analyses, we conducted sensitivity analyses using secondary summary indices of cortisol reactivity, including AUCi, peak cortisol, and ΔCortisol (peak–baseline). Across all chronic stress exposures, the direction of effects for these indices was consistent with the main time × group interactions observed in the primary LMMs. Participants reporting higher levels of financial strain, social isolation, negative life events, or perceived stress generally exhibited greater cumulative cortisol output (AUCi), higher posttask peak cortisol levels, and larger cortisol increases from baseline (Supplementary Table S2). Although some between-person comparisons did not reach statistical significance (two-tailed *P* < 0.05), this may partly reflect the limited statistical power of these aggregate summary analyses in the modest sample (*N* = 60). Nevertheless, the overall pattern of findings supports the primary results, indicating heightened HPA axis reactivity among individuals with greater chronic stress exposure. To address the potential effects of skewness in the cortisol level outcomes, we re-ran the primary analyses using log-transformed cortisol levels. The results were consistent with the primary findings, showing no meaningful changes (see Supplementary Tables S3 and S4).

## Discussion

In this study, we examined the association between patient-reported data on social stressors and cortisol responses among African American patients with breast cancer. We found that the magnitude of cortisol response is moderated by higher levels of social stressors. Patients who experienced greater financial strain, social isolation, negative life events, and perceived stress had higher peak cortisol levels during a laboratory-based stressor than their counterparts. This suggests that African American patients with breast cancer with more exposure to social stressors may experience dysregulated HPA axis hyperactivity compared with those who have less exposure to social stressors.

In other studies, the social stressors we measured adversely affected survivorship quality and were associated with cancer processes and outcomes (8, 12, 13). Recent cross-sectional research examined the relationship between immune responses and exposure to social stressors (13); however, immune function and immunologic responses in the tumor microenvironment are activated by the HPA axis response and exposure to stress hormones (11). Cortisol also exerts complex effects on cancer cells and tumor growth through interaction with glucocorticoid receptors (28–30). Across all of the social stressors measured in this study, patients who reported greater exposure to social stressors had higher peak cortisol reactivity compared with those who reported lower exposure. Exposure to social stressors has not been examined in previous research that evaluated stress reactivity among breast cancer survivors (31); the consistent pattern of dysregulated cortisol responses observed across all the social stressors we measured underscores the importance of screening for these issues using patient-reported data.

Apart from negative life events, the social stressors we measured are components of the SDOH screening frameworks and tools used by health care systems to identify patients who have unmet needs (32). Previous research has shown that SDOH screening is likely to be completed as part of patient intake and rooming (33), and evidence is emerging about the feasibility of completing SDOH screening as part of these workflows. For instance, findings from a mixed-methods qualitative study demonstrated that screening patients for SDOH using an EHR-based tool was feasible based on the percentage of patients who were screened and the median clinic visit time (34). In contrast, Brewster and colleagues (35) found that only 27% of practices included in the National Survey of Healthcare Organizations and Systems reported screening for social risk factors. Relatedly, less than 1% of patients who completed an outpatient visit had SDOH documented in the EHR using Z-codes, and there was incongruence between the social need recorded using the Z-codes and those identified by providers (36).

Clearly, cumulative patient-reported data on social stressors are important to understand the lived experiences of patients with cancer comprehensively and identify those who have unmet needs. Consistent with this, recent investigations have examined the association between the number of social risk factors and cancer screening (37). However, the collection of actionable data on social risk factors using sustainable strategies should be prioritized to ensure and enhance the impact of investing resources to collect, analyze, and monitor the distribution of social risk factors and evaluate the outcomes of socially informed interventions on health care outcomes and quality. We were able to obtain cumulative data on social risk factors from patients as part of a research protocol that examined the association between these issues and physiologic stress responses. This approach may not be possible in all clinical settings and may have unintended adverse consequences in health care settings in which resources are not available to mitigate the social risk identified by SDOH screening. Thus, it may be important to prioritize the collection of social risk data depending on the clinical context (e.g., primary or oncology care) and/or the type of cancer care being delivered. Our findings demonstrate that data on social risk factors related to financial strain, perceived risk, social isolation, and negative life events could be tailored to the clinical context or intervention while also providing insights into an underlying biological mechanism that is involved in disease processes. That is, the total number of instruments that are used to screen patients for SDOH could be reduced without sacrificing the relevance of the data to multilevel drivers (e.g., biological and social) of cancer care outcomes. This is because the magnitude of differences in cortisol levels between high– and low–social stress exposure groups was relatively consistent among the risk factors measured. Screening patients for SDOH using a reduced number of instruments may have advantages in terms of reducing patient burden, increased integration of SDOH screening into clinical operations, and greater alignment of SDOH screening with the clinical focus of cancer care.

In considering the results of this study, some limitations should be noted. First, the generalizability of our findings is limited to African American patients with breast cancer who were recruited from one NCI-designated cancer center. Furthermore, measuring stress response using a single biomarker and the lack of a longitudinal assessment of stress reactivity did not allow us to capture the entire complexity of the physiologic stress response over time. We did not collect data on overall or progression-free survival for the patients, and racial/ethnic group comparisons in stress reactivity were not conducted. Information on menstrual cycle phase, hormonal contraceptive or endocrine therapy use, sleep/wake timing, and recent caffeine or nicotine intake was not systematically collected, although given the within-subject design, we believe the primary impact of such factors is minimized. The internal consistency of the PSS in this sample was moderate (Cronbach α = 0.76), suggesting some inter-item variability in stress perception, which may contribute to nondifferential measurement error and modest bias associations with physiologic stress markers toward the null. Undoubtedly, African American women continue to experience greater mortality from breast cancer and have had limited representation in previous research that examined stress reactivity among breast cancer survivors (37).

The use of a standardized and validated laboratory method to activate the HPA axis response is an important strength of our study that was enhanced by obtaining patient-reported data on exposure to social stressors. Together, patient self-reported data on social stressors and objective biomarker data on cortisol changes among African American patients with breast cancer provide innovative and novel empirical data about stress reactivity and the likelihood of a dysregulated stress response based on exposure to social stressors. Our findings suggest that African American women who are breast cancer survivors with persistent exposure to stress-inducing factors may experience heightened cortisol responses to acute stress, potentially increasing their cumulative lifetime exposure to elevated cortisol. Longitudinal studies are needed to investigate the potentially causal pathways linking multilevel social stress exposures with HPA axis regulation and downstream biological processes relevant to breast cancer survivorship. Interventions targeting modifiable behavioral pathways, such as cognitive–behavioral stress management, could help determine whether improvements in these domains translate into more adaptive cortisol profiles and better survivorship outcomes.

### Conclusions

In conclusion, our findings provide novel empirical evidence that social risk factors moderate cortisol reactivity among African American breast cancer survivors. Our findings show that patient-reported data on social stressors related to financial strain, social isolation, perceived stress, and negative life events could be used to increase the precision of SDOH screening, enhance the identification of patients who are likely to have a dysregulated stress response, and tailor patient referrals to social services and public health agencies to address unmet needs. Evidence-based interventions can only be implemented with precision when whole-person health data are collected at each clinical visit. SDOH screening that is aligned with social risk factors that are also associated with biological mechanisms involved in cancer risk and outcomes will enhance cancer care quality and minimize barriers that contribute to cancer health disparities. Future studies that integrate SDOH data with other biomarkers may enhance our understanding of the full range of factors that influence disparities in outcomes among patients with breast cancer, paving the way for novel combination interventions that address social and molecular determinants.

## Supplementary Material

Figure S1 Process model of study procedures

Figure S2 Individual spaghetti plot of cortisol level across timepoints

Table S1 Associations between covariates and cortisol levels

Table S2 Summary of supplemental cortisol reactivity indices (AUCi, Peak cortisol, and ΔCortisol) by chronic stress exposures

Table S3 Associations between assessment time points and log-transformed cortisol levels

Table S4 Interaction effects test for log-transformed cortisol levels

## Data Availability

The data dictionary for this study and summary information about sample characteristics are available upon written request to the corresponding author.
