# Supplementary material for: Examining the Intersection between Drivers of Disparities: Social Determinants and Stress Reactivity in African American Breast Cancer Survivors
Source: Cancer Res Commun. 2026 Mar 30;6(3):698–705. doi: 10.1158/2767-9764.CRC-25-0388 (PMC13033974; doi:10.1158/2767-9764.CRC-25-0388)
Supplement: Figure S1 — Process model of study procedures [file crc-25-0388_figure_s1_suppsf1.pdf]

## Figure S1. Process model[a]

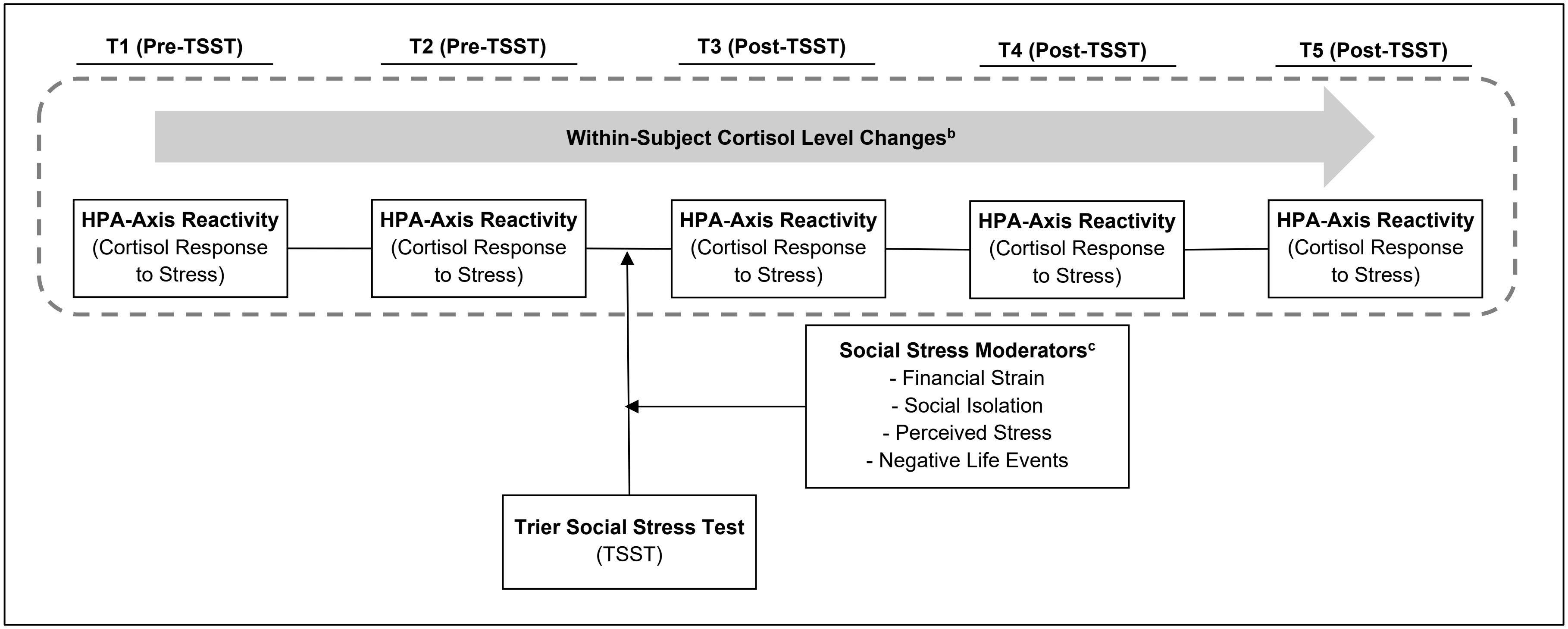


[a] Analytic sample N=60 (African American breast cancer survivors.

[b] Linear mixed effect repeated-measures regression models to examine within-subject changes in cortisol levels across pre- and post-TSST (T1-T5).

[c] Moderation effects of social stress exposures on changes in cortisol levels across T1-T5 (i.e., Interaction effects of time X each social stress moderator).
