# Supplementary material for: Examining the Intersection between Drivers of Disparities: Social Determinants and Stress Reactivity in African American Breast Cancer Survivors
Source: Cancer Res Commun. 2026 Mar 30;6(3):698–705. doi: 10.1158/2767-9764.CRC-25-0388 (PMC13033974; doi:10.1158/2767-9764.CRC-25-0388)
Supplement: Figure S2 — Individual spaghetti plot of cortisol level across timepoints [file crc-25-0388_figure_s2_suppsf2.pdf]

Figure S2. Individual spaghetti plot of cortisol level across timepoints[a,b]

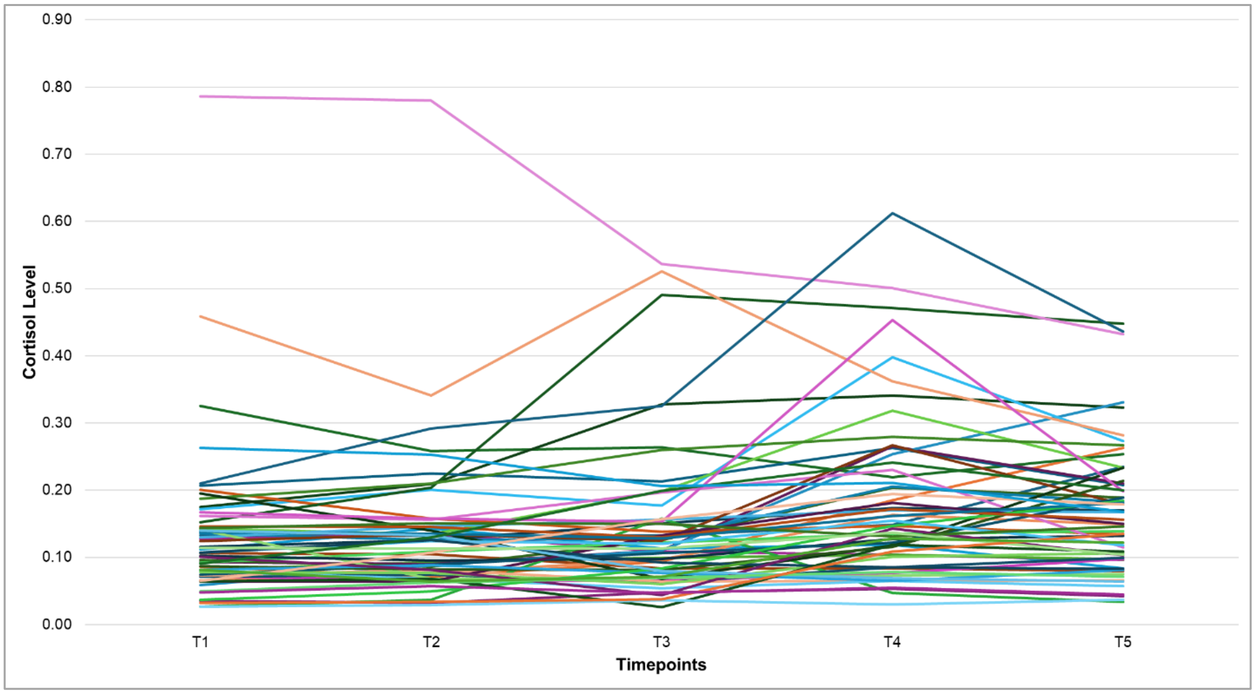

[a] Analytic sample participants N = 60 (Total observation N = 300).
[b]T1 and T2 were assessed before TSST tasks and T3, T4, and T5 were assessed after TSST tasks.
