# Supplementary material for: Examining the Intersection between Drivers of Disparities: Social Determinants and Stress Reactivity in African American Breast Cancer Survivors
Source: Cancer Res Commun. 2026 Mar 30;6(3):698–705. doi: 10.1158/2767-9764.CRC-25-0388 (PMC13033974; doi:10.1158/2767-9764.CRC-25-0388)
Supplement: Table S1 — Associations between covariates and cortisol levels [file crc-25-0388_table_s1_suppst1.pdf]

Table S1. Associations between covariates and cortisol levels[a]

Regressors	b (95%CI)[b]	P
Age, y	0.003 (0.001, 0.005)	.04
Marital status		
Not married	REF	--
Married	0.008 (-0.036, 0.052)	.71
Education level		
≤High School	REF	--
≥Some College	0.028 (-0.033, 0.089)	.37
Employment Status		
Employed	REF	--
Retired	-0.004 (-0.060, 0.051)	.87
Not Employed	-0.029 (-0.089, 0.031)	.34
Income Level		
≥$35,000	REF	--
<$35,000	-0.064 (-0.157, 0.028)	.17
Time since diagnosis		
≤Two Years	REF	--
>Two Years	0.048 (-0.013, 0.108)	.12
Stage		
Ib or lower	REF	--
IIa or greater	-0.047 (-0.101, 0.007)	.09
Financial strain[c]	0.002 (-0.079, 0.081)	.98
Social isolation[c]	-0.008 (-0.054, 0.038)	.72
Perceived stress[c]	0.036 (-0.004, 0.076)	.08
Negative events[c]	-0.019 (-0.087, 0.050)	.59

[a] Participants N=60 (Total observation points N = 300).
[b] Linear mixed effect repeated-measures regression models for cortisol level outcome including time and covariates (see adjusted model in Table 2).
[c] Each moderator was coded as 0 (Low) or 1 (High) using median split.
