# Supplementary material for: Examining the Intersection between Drivers of Disparities: Social Determinants and Stress Reactivity in African American Breast Cancer Survivors
Source: Cancer Res Commun. 2026 Mar 30;6(3):698–705. doi: 10.1158/2767-9764.CRC-25-0388 (PMC13033974; doi:10.1158/2767-9764.CRC-25-0388)
Supplement: Table S2 — Summary of supplemental cortisol reactivity indices (AUCi, Peak cortisol, and ΔCortisol) by chronic stress exposures [file crc-25-0388_table_s2_suppst2.pdf]

Table S2. Summary of supplemental cortisol reactivity indices (AUCi, Peak cortisol, and ΔCortisol) by chronic stress exposures[a]

Chronic stress exposure status	AUCi[b]		Peak Cortisol[c]		ΔCortisol[d]	
	M (95%CI)[e]	P	M (95%CI)[e]	P	M (95%CI)[e]	P
Financial strain[f]						
High	1.23 (0.85, 1.62)	.003[g]	0.21 (0.17, 0.26)	.09	0.09 (0.04, 0.13)	.13
Low	0.45 (0.12, 0.78)		0.17 (0.13, 0.20)		0.04 (0.02, 0.08)	
Social isolation[f]						
High	1.32 (0.87, 1.77)	.13	0.22 (0.17, 0.27)	.10	0.10 (0.06, 0.13)	.03[g]
Low	0.81 (0.25, 1.36)		0.17 (0.13, 0.21)		0.04 (0.01, 0.07)	
Negative life events[f]						
High	1.14 (0.83, 1.46)	.01[g]	0.22 (0.17, 0.28)	.18	0.08 (0.04, 0.11)	.19
Low	0.52 (0.13, 0.90)		0.18 (0.13, 0.22)		0.05 (0.02, 0.09)	
Perceived stress[f]						
High	1.29 (0.91, 1.66)	.001[g]	0.23 (0.18, 0.29)	.03[g]	0.09 (0.06, 0.13)	.02[g]
Low	0.33 (0.02, 0.65)		0.16 (0.13, 0.20)		0.04 (0.01, 0.07)	

[a] Participants N=60.
[b] AUCi = area under the curve with respect to increase (baseline-adjusted total cortisol output across post-task timepoints).
[c] Peak cortisol = the maximum cortisol value measured at T3–T5 (post-task timepoints).
[d] ΔCortisol = peak minus baseline cortisol.
[e] Estimated marginal means adjusting for time-invariant covariates presented in the adjusted model in Table 2
[f] Each moderator was coded as 0 (Low) or 1 (High) using a median split.
[g] Statistically significant after Benjamini-Hochberg corrections for multiple testing to control false-discovery rate at .05 (based on 2-tailed corrected P-value).
