# Supplementary material for: Examining the Intersection between Drivers of Disparities: Social Determinants and Stress Reactivity in African American Breast Cancer Survivors
Source: Cancer Res Commun. 2026 Mar 30;6(3):698–705. doi: 10.1158/2767-9764.CRC-25-0388 (PMC13033974; doi:10.1158/2767-9764.CRC-25-0388)
Supplement: Table S3 — Associations between assessment time points and log-transformed cortisol levels [file crc-25-0388_table_s3_suppst3.pdf]

Table S3. Associations between assessment time points and log-transformed cortisol levels [a]

	Unadjusted model [b]		Adjusted model [c]	
Time[d]	Exp(β)[e] (95%CI)	P	Exp(β)[e] (95%CI)	P
T1	REF	--	REF	--
T2	1.033 (0.982, 1.086)	.12	1.038 (0.985, 1.095)	.16
T3	1.084 (0.962, 1.221)	.19	1.089 (0.958, 1.237)	.19
T4	1.399 (1.227, 1.595)	<.001[f]	1.435 (1.251, 1.646)	<.001[f]
T5	1.345 (1.175, 1.539)	<.001[f]	1.367 (1.186, 1.575)	<.001[f]

[a] Participants N=60 (Total observation points N = 300).

[b] Linear mixed effect repeated-measures regression models for cortisol level outcome including time with random intercept.

[c] Linear mixed effect repeated-measures regression models for cortisol level outcome including time regressor and time-invariant covariates (i.e., age, marital status, education level, employment status, income level, time since diagnosis, stage, financial strain, social isolation, negative life events, and perceived stress).

[d] T1 and T2 were assessed before the TSST tasks and T3, T4, and T5 were assessed after TSST tasks.

[e] exponentiated coefficients for log-transformed continuous cortisol level outcome.

[f] Statistically significant after Benjamini-Hochberg corrections for multiple testing to control false-discovery rate at .05 (based on 2-tailed corrected P-value).
