# Supplementary material for: Examining the Intersection between Drivers of Disparities: Social Determinants and Stress Reactivity in African American Breast Cancer Survivors
Source: Cancer Res Commun. 2026 Mar 30;6(3):698–705. doi: 10.1158/2767-9764.CRC-25-0388 (PMC13033974; doi:10.1158/2767-9764.CRC-25-0388)
Supplement: Table S4 — Interaction effects test for log-transformed cortisol levels [file crc-25-0388_table_s4_suppst4.pdf]

Table S4. Interaction effects test for log-transformed cortisol levels [a]

Interaction effect test [b]				
**Interaction term**	**χ2/df**	**P**		
Time X Financial strain	10.02/4	.04		
Time X Social isolation	9.98/4	.04		
Time X Negative life events	9.67/4	.04		
Time X Perceived stress	10.81/4	.03		
**Post-hoc multigroup analysis 1 – Financial strain[c]**				
	**Low financial strain**		**High financial strain**	
Time[b]	**Exp(β) (95%CI)**	**P**	**Exp(β) (95%CI)**	**P**
T1	REF	--	REF	--
T2	1.048 (0.984, 1.116)	.14	1.029 (0.965, 1.098)	.38
T3	1.073 (0.821, 1.402)	.61	1.089 (0.953, 1.244)	.21
T4	1.249 (0.985, 1.583)	.07	1.453 (1.245, 1.696)	<.001[e]
T5	1.217 (0.973, 1.522)	.10	1.446 (1.226, 1.704)	.001[e]
**Post-hoc multigroup analysis 2 – Social isolation[c]**				
	**Low social isolation**		**High social isolation**	
Time[b]	**Exp(β) (95%CI)**	**P**	**Exp(β) (95%CI)**	**P**
T1	REF	--	REF	--
T2	1.071 (0.972, 1.156)	.13	0.986 (0.906, 1.073)	.98
T3	1.125 (0.976, 1.298)	.59	1.039 (0.855, 1.263)	.70
T4	1.249 (0.991, 1.577)	.05	1.456 (1.220, 1.737)	<.001[e]
T5	1.178 (0.961, 1.443)	.13	1.424 (1.212, 1.672)	<.001[e]
**Post-hoc multigroup analysis 3 – Negative life events[c]**				
	**Low negative life events**		**High negative life events**	
Time[b]	**Exp(β) (95%CI)**	**P**	**Exp(β) (95%CI)**	**P**
T1	REF	--	REF	--
T2	1.052 (0.977, 1.134)	.17	1.017 (0.949, 1.090)	.63
T3	1.027 (0.859, 1.228)	.77	1.136 (0.969, 1.331)	.12
T4	1.264 (1.005, 1.592)	.04	1.448 (1.171, 1.792)	<.001[e]
T5	1.203 (0.964, 1.503)	.06	1.499 (1.194, 1.883)	<.001[e]
**Post-hoc multigroup analysis 4 – Perceived stress[c]**				
	**Low perceived stress**		**High perceived stress**	
Time[b]	**Exp(β) (95%CI)**	**P**	**Exp(β) (95%CI)**	**P**
T1	REF	--	REF	--
T2	1.039 (0.978, 1.105)	.21	1.018 (0.931, 1.113)	.69
T3	1.094 (0.954, 1.256)	.20	1.054 (0.828, 1.340)	.67
T4	1.163 (0.842, 1.612)	.09	1.576 (1.240, 2.003)	<.001[e]
T5	1.242 (0.947, 1.619)	.15	1.490 (1.251, 1.774)	<.001[e]

[a] Participants N=60 (Total observation N = 300).
[b] Linear mixed effect repeated-measures regression models for cortisol level outcome include time and the interaction term of time X each moderator (i.e., financial strain, social isolation, negative life events, perceived stress), adjusting for time-invariant covariates presented in the adjusted model in Table 2. Each interaction effect for individual moderator was separately tested. For significant interaction terms based on the Wald Chi-square/df test, post-hoc multigroup analyses were conducted to examine the association between time and cortisol level outcome, stratified by the moderator status.
[c] Each moderator was coded as 0 (Low) or 1 (High) using median split.
[d] T1 and T2 were assessed before TSST tasks and T3, T4, and T5 were assessed after TSST tasks.
[e] Statistically significant after Benjamini-Hochberg corrections for multiple testing to control false-discovery rate at .05 (based on 2-tailed corrected P-value).
